# The role of management in providing safe eye care delivery

**Published:** 2021-07-20

**Authors:** Ushalini Rasiah, Ravindran D Ravilla, Thulasiraj D Ravilla

**Affiliations:** 1 Quality Manager: Aravind Eye Care System, Madurai, India; 2 Chairman & Director: Quality Aravind Eye Care System, Madurai, India.; 3 Executive Director: Lion’s Aravind Institute of Community Ophthalmology (LAICO), Madurai, India. Director-Operations: Aravind Eye Care System, Madurai, India.

**In order to deliver high quality, safe eye care to patients and ensure a positive, care-giving attitude among staff members, it is vital that leaders and managers recognise and prioritise patient and staff safety.**

The role of management in ensuring the safety of patients is critical, but it is often overlooked in safety programmes for eye care providers. Health care leadership and management are responsible for creating an environment that protects patients and staff members from avoidable harm and reduces errors in clinical settings. Creating this environment involves:

- Being clear about the safety goals in every area of work
- Creating well-defined processes or standard operating procedures (SOPs) to achieve these goals
- Identifying the staff member(s) responsible for each process
- Training staff members in the processes and SOPs
- Providing clarity on what incidents to report
- Setting up systems for continually refining processes and reviewing SOPs
- Identifying individuals who will lead and can be held accountable for maintaining safety in a given work area.

**How safe is eye care?**

It may be tempting to think of eye care as relatively safe, compared to other medical specialties. However, a recent study of medical errors (adverse events) in US Veterans Health Administration (VHA) medical centres from 2010–2017 showed that the highest number of reported errors or adverse events occurred in ophthalmology (72) followed by dentistry (30) and anaesthesiology (28).^1^ Reducing eye care related adverse events is a challenge. In many settings, patient volumes and time pressures are high. The most common eye procedure – cataract surgery – involves many steps, with many opportunities for error: starting with documenting several measurements using multiple equipment (during biometry), then sourcing a non-expired intraocular lens of the correct power, followed by carrying out safe surgery on the correct patient, in the correct eye.^2^^,^^3^

An organisation that addresses safety (and any errors that may occur) in an open and transparent way demonstrates to staff members and the public that it values their wellbeing.^4^ This encourages staff members to prioritise a culture of safety, knowing they have the support of their managers. It also makes the organisation more efficient and the care process more effective. With this in mind, Aravind Eye Hospitals has developed a systematic approach to providing safer eye care delivery by adopting the following principles.

**Defining safety goals.** Safety goals must be defined at an organisational level and adapted to suit individual departments or settings. The formal efforts to integrate patient safety in Aravind eye hospitals was initiated in 2007–08. Initially, Aravind adopted the 2009 Joint commission (USA) hospital patient safety goals,^5^ which staff members struggled to relate to their eye care work. Realising this, and based on the incidents that were reported, the authors adapted the Joint Commission (USA) objectives and developed the safety goals in Figure 1, specific to eye care, in 2013–2014. It was felt to be equally important to have specific goals at department level (Figure 2, overleaf).

**Figure 1:**
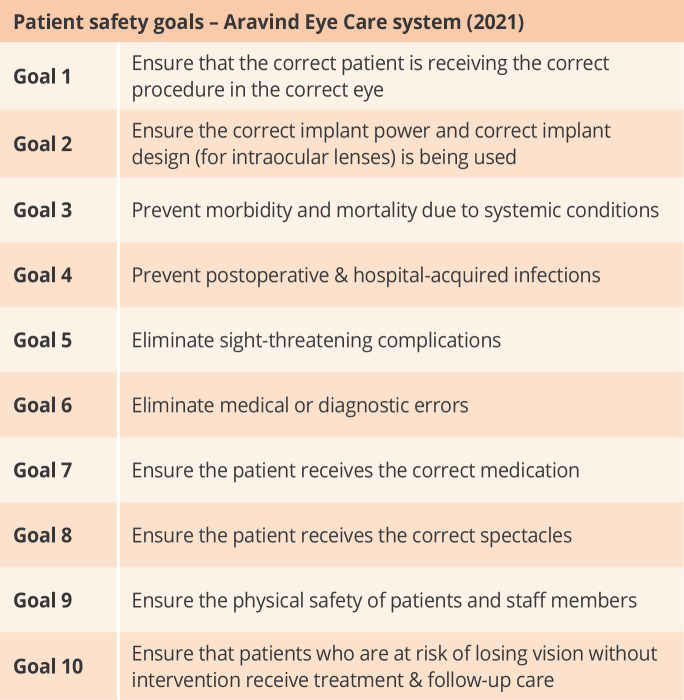
Eye care safety goals: organisation level

**Figure 2:**
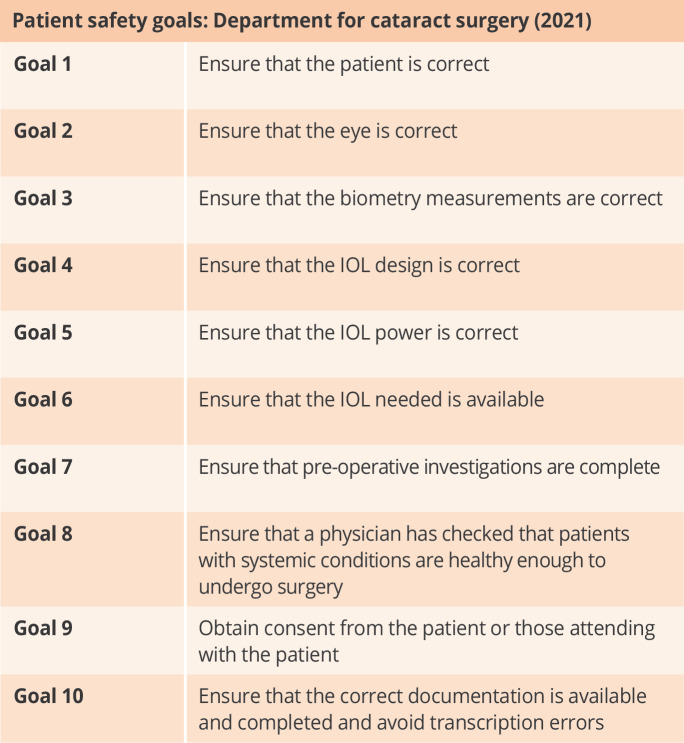
Department-specific safety goals focusing on patient safety. E.g., *Counselling for cataract surgery*

**Creating systems that enhance patient safety** is crucial as errors often happen due to failures in the system. The causes go beyond the individuals who may have made mistakes. Good safety systems ensure standardisation of procedures, specific steps to ensure safety protocols, appropriate delegation of work to the right personnel, and checklists at critical points in the patient’s journey. Developing and refining systems, with full participation of staff members, will streamline the work and reduce risk.

**Standardising and continuously improving work processes** progressively reduces risk. Senior clinical staff and managers are responsible for creating standard operating procedures (SOPs) and risk assessments for routine clinical activities and administrative tasks (e.g., booking follow-up appointments). Managers must also ensure that every member of staff is trained in these SOPs and competent to deliver them. SOPs and risk assessments should be made easily accessible to staff members in the form of posters or quick reference guides which are regularly reviewed and updated.

**Choosing suitable equipment and ensuring all staff members are trained appropriately.** Routine eye care involves the use of both basic and highly technical instruments and equipment. All staff members who use instruments and equipment should be appropriately trained beforehand, certified and have access to supporting documentation (SOPs, user manuals, and risk assessments).

**Building an organisational structure and process for safety** is crucial. It is important to develop this structure, at both leadership and operational level. For example, staff members can be assigned the role of ‘safety champions’. The main role of a ‘safety champion’ is to model safe practices and behaviour. A champion is selected from each group or type of eye care worker; they then form a team that meets regularly to contribute to the development and implementation of safe practice in all areas. Another approach is to invite staff members and managers to share ideas and experiences to improve processes in eye care and to mitigate risks through sharing lessons learned. This must be done in a supportive and positive way, particularly when discussing errors or difficulties that may have occurred. This encourages staff to learn from previous mistakes and understand how to avoid them in future. Such discussions provide an opportunity to understand the magnitude of errors, their frequency, and what contributes to them.

**Creating a culture of reporting errors and near misses** is a core element of good clinical practice. Early reporting of errors improves staff and patient safety and makes it possible to investigate and address the root causes of the error.^6^ Ideally, a reporting system should make it possible for staff members to report incidents anonymously – or ‘near misses’ without identifying themselves, the patient, or the staff members involved. This allows others to learn from the situation without fear and enhances the commitment of staff members towards safer care.^7^ There is no universally accepted way to report errors, but it is possible to create a simple form to record essential details such as time and place, people involved, description of the error, and the possible circumstances that led to the error.

**Incident reporting system**

Aravind originally used a paper-based reporting system, with low reporting rates. A computer-based, online reporting system was set up 8 years ago, and since then more than 16,500 events have been reported. The authors believe this system to be more successful because it is now easier for anyone to report a safety-related incident or near miss anonymously.

The value of any reporting system lies in how useful it is in helping to avoid future incidents. Each incident reported using this system is therefore brought to the attention of the chief medical officer and quality manager instantly, via an automatic e-mail. These senior staff members, who are empowered to address the root causes of safety incidents, can also use the system to carry out detailed analyses and generate actionable reports.

**Supporting staff wellbeing.** As a service sector, it is vital that leaders and managers recognise the importance of ensuring the wellbeing of staff members. Fatigue levels, inadequate training and a stressful work environment can contribute to human errors and affect compliance with SOPs and risk assessments. A deeper understanding of these factors, and addressing them, will lead to safer practices.

One of the factors that contribute to employee stress is unfair treatment or harassment. Managers can establish a culture of respect and dignity in the workplace by:

- Taking the time to get to know staff members and the challenges they face
- Treating everyone with respect (i.e., modelling appropriate behaviour)
- Having an ‘open door’ policy that allows any staff member to make a suggestion, ask a question, or raise a concern.

Other issues affecting staff members’ stress levels and health at work can include inadequate rest breaks, a lack of access to healthy food and beverages, and scheduling difficulties (e.g., if a person is given too many night shifts in a short space of time).

Finally, **ongoing education programmes** for staff members and managers that focus on safety are essential to continually build awareness and promote safe practices.

1. **Constant monitoring** and review is vital to ensure the efficacy of safe processes. It is important to do root cause analysis of all incidents and near misses and discuss them as a group, both for further refinement and for reinforcing the value of following set safety protocols.
2. **Constant reinforcement** by the organisational leadership of their commitment to protect staff and patients is important. Safety is not a destination, but a continuous process.

## Conclusion

Developing a sustained safety programme is challenging; it must start with the commitment of managers and leaders. Leaders who are passionate about safety should establish the process for achieving the safety outcomes in both clinical and non-clinical areas, build local leadership to take ownership at operational level, foster a safety culture, prioritise safety through proper communication with their teams, and provide appropriate resources to build practices that enhance safety.
